# Protein photodegradation in the visible range? Insights into protein photooxidation with respect to protein concentration

**DOI:** 10.1016/j.ijpx.2022.100155

**Published:** 2022-12-27

**Authors:** Elena Hipper, Florian Lehmann, Wolfgang Kaiser, Göran Hübner, Julia Buske, Michaela Blech, Dariush Hinderberger, Patrick Garidel

**Affiliations:** a Institute of Chemistry, Martin-Luther-Universität Halle-Wittenberg, von-Danckelmann-Platz 4, 06120 Halle, Germany; b Boehringer Ingelheim Pharma GmbH & Co. KG, Innovation Unit, PDB-TIP, Birkendorfer Strasse 65, 88397 Biberach an der Riss, Germany; c Boehringer Ingelheim Pharma GmbH & Co. KG, Innovation Unit, ADB, Birkendorfer Strasse 65, 88397 Biberach an der Riss, Germany

**Keywords:** Monoclonal antibody, Visible light exposure, Photooxidation, Polysorbate 20, PS20, Electron paramagnetic resonance, EPR

## Abstract

Visible light (400–800 nm) can lead to photooxidation of protein formulations, which might impair protein integrity. However, the relevant mechanism of photooxidation upon visible light exposure is still unclear for therapeutic proteins, since proteinogenic structures do not absorb light in the visible range. Here, we show that exposure of monoclonal antibody formulations to visible light, lead to the formation of reactive oxygen species (ROS), which subsequently induce specific protein degradations. The formation of ROS and singlet oxygen upon visible light exposure is investigated using electron paramagnetic resonance (EPR) spectroscopy. We describe the initial formation of ROS, most likely after direct reaction of molecular oxygen with a triplet state photosensitizer, generated from intersystem crossing of the excited singlet state. Since these radicals affect the oxygen content in the headspace of the vial, we monitored photooxidation of these mAb formulations. With increasing protein concentrations, we found (i) a decreasing headspace oxygen content in the sample, (ii) a higher relative number of radicals in solution and (iii) a higher protein degradation. Thus, the protein concentration dependence indicates the presence of higher concentration of a currently unknown photosensitizer.

## Graphical abstract

**Figure:**
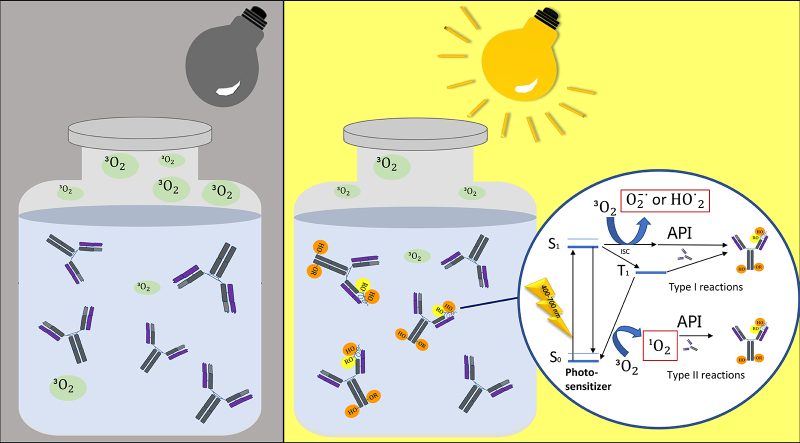

## 1 Introduction

Monoclonal antibodies (mAbs) are highly complex active pharmaceutical ingredients (API) that require special care during processing due to their higher-order structure. This aspect is important to maintain the biological activity. Oxidation of susceptible amino acids might induce structural change of mAbs, which can result in aggregation, particle formation and reduced biological activity (Boll et al., 2017; Fradkin et al., 2014; Gupta et al., 2021; Narhi et al., 2015; Corvari et al., 2015). Since these proteins are exposed to light at different stages throughout development, e.g. during manufacturing, processing, shipping and drug administration, photostability testing is an integral part of stress testing of new drugs (International Comission on Harmonization, 1995). Many therapeutic proteins have been found photosensitive when exposed to ICH Q1B light conditions composed of visible and ultraviolet (UV) light (Dalsgaard et al., 2007; Gomyo and Fujimaki, 1970; Roy et al., 2009) (ICH Q1B: illumination not less than 1.2 million lux hours and integrated UV energy of not less than 200 W·h/m^2^). However, during normal laboratory and manufacturing processes proteins are exposed to visible light with wavelengths predominantly >400 nm (illuminance around 43–260·10^3^ lx·h) (Sreedhara et al., 2016) and to a much smaller extent to UV light in the λ = 350–400 nm range (irradiance between 1.6 and 10 W·h/m^2^, but typically 0.3–3 W·h/m^2^ UV light) (Sreedhara et al., 2016). In general, chromophore residues must absorb light in this wavelength range for a photoreaction to take place (Winchester, 1990). Although mAbs should not absorb light in the visible range (Sreedhara et al., 2016; Davies and Truscott, 2001; Luis et al., 2018; Mantha et al., 2020), an increased degradation upon exposure to visible light was reported (Sreedhara et al., 2016; Luis et al., 2018; Du et al., 2018; Kaiser et al., 2021; Kerwin and Remmele, 2007; Mallaney et al., 2014). Hence, visible light in the spectral range of λ = 400–800 nm might degrade proteins indirectly. In the literature, some impurities from the manufacturing process of mAbs are described as photoactive compounds (photosensitizer) in these wavelength ranges, such as riboflavin impurities from cell culture (Schöneich, 2020) or catalytic amounts of iron present in pharmaceutical excipients (Subelzu and Schöneich, 2020; Subelzu and Schöneich, 2021; Stroop et al., 2011; Ha et al., 2002). Polysorbate (PS) in particular has been suggested as a possible source of photoactive substances (Prajapati et al., 2020; Agarkhed et al., 2013; Singh et al., 2012). Polysorbate is a commonly used excipient in biologics to stabilize proteins from surface adsorption, reduce protein particle formation and denaturation at the liquid/air interface (Mahler et al., 2010; Tomlinson et al., 2015; Singh et al., 2017; Garidel et al., 2021). Since intact polysorbate 20 itself does not absorb light in the visible region (Wuelfing et al., 2006), the photosensitizing compound(s) in the solution of polysorbate 20 is/are still unclear. In drug product formulations consisting of the mAb and excipients, a non-proteinogenic photosensitizer that is present may absorb visible light (Schöneich, 2020; Stroop et al., 2011; Prajapati et al., 2022) and as a consequence thereof, may degrade proteins by the following potential mechanism. The excited singlet state of the photosensitizer can undergo several processes, including intersystem crossing (ISC) to a triplet state. The formation of the triplet state is the central step towards photooxidation. The photosensitizer in the longer-lived (e.g. for oxygen, the half-life is in the μs range instead of ns) triplet state can undergo energy or electron transfer reactions, which are either denoted as type I (electron transfer) or type II (energy transfer) reactions (Baptista et al., 2017). A photosensitizer in a triplet state may react with substrates such as the ground state of oxygen (triplet state molecule). Oxygen quenches the triplet state and can form activated oxygen intermediates, either substrate radical anions (type I) or singlet oxygen (type II). These activated oxygen species may interact with proteins and induce a degradation via oxidation. The amino acids most susceptible to oxidation are Trp, His, Met, Tyr, and Cys (Matheson et al., 1975).

The present study aims investigating the effects of visible light exposure on the radical formation in mAb formulations, followed by subsequent oxygen content depletion in the vial's headspace and ultimately analysis of protein degradation. The first part of the study describes the decrease in oxygen content in the headspace of the vial due to the generation of radicals. There is a correlation between decreased oxygen content and protein particle formation dependent on the protein concentration. Since drug product formulations contain the API and excipients such as polysorbate, the photostability of mAb formulations was investigated in the presence of different PS concentrations. The second part of the study is focused on the use of TEMPOL (4-hydroxy-2,2,6,6-tetramethylpiperidinyl-1-oxyl) as a radical quencher monitored using electron paramagnetic resonance (EPR) spectroscopy. We obtained direct evidence that free radicals are formed when the investigated mAb formulations are subjected to illumination with visible light. With these methods, we were able to determine increased oxygen depletion, protein degradation and radical formation as a function of increased protein concentration. This suggests that a potential photosensitizer(s) is present in the protein solutions. However, it is still unclear which photosensitizer underlies the generation of reactive oxygen species.

## 2 Experimental section

### 2.1 Materials, reagents and sample preparation

#### 2.1.1 Materials and reagents

Three recombinant humanized monoclonal antibodies (termed as mAb-Ι, ΙΙ and ΙΙΙ) provided by Boehringer Ingelheim GmbH & Co.KG (Biberach an der Riß, Germany) were used for the light studies. The three mAbs are of isotype IgG1, have an average molecular weight of approximately 150 kDa and were produced by mammalian cell culture technology using a Chinese hamster ovary cell line (Pisch-Heberle et al., 2008). All mAbs were purified prior to light studies utilizing Protein A chromatography. High purity (HP) quality grade polysorbate 20 is obtained from Croda (East Yorkshire, United Kingdom). The following studies were performed uniquely with PS20 HP. In samples containing polysorbate (PS20 HP), the concentration was adjusted by spiking a 100 mg·ml^−1^ (*w*/*v*) PS20 HP stock solution in water into the formulation buffer to achieve the desired PS20 HP concentration. All chemicals were of analytical grade and were purchased from Sigma-Aldrich such as 4-hydroxy-2,2,6,6-tetramethylpiperidinyl-1-oxyl (TEMPOL). All samples were protected from light during sample handling and storage.

#### 2.1.2 Sample preparation for light exposure and oxygen content measurement

The different concentrations of mAb-Ι, ΙΙ and ΙΙΙ were filled in 2/4 ml glass type 1 vials (Schott AG, Germany) closed with the Fluro Tec® rubber stopper (Daikyo, Japan) prior to light exposure. To monitor a high depletion of oxygen content, the following set up was chosen: the fill volume of the formulation was 3.9 ml and the volume of the air headspace estimated to be 0.1 ml. All mAbs were formulated in 50 mM phosphate buffer at pH 6.2 with 159 mM trehalose. The same formulation was used for all experiments and mAbs to exclude formulation dependent effects. Light exposure was performed in a calibrated light chamber (PharmaEvent, Weiss Technik) at 25 °C and 60 % relative humidity (RH). The emission spectrum of the bulbs in the chamber are depicted in the supplementary material section (S1). The vials were placed upside-down to reflect worst-case conditions. The light source (Planistar 41–57-SLED-1-VD2-W light sheet) was located directly above the sample solution, and thus were exposed to visible light (λ = 400–800 nm) with an intensity of 10′000 lx for 0, 24, and 72 h. The corresponding dark control samples were stored upside-down in light-tightened containers and placed in the photostability chamber using the same conditions. The oxygen contents were measured directly after light exposure. After oxygen content measurement, the samples were stored in light-tight containers at 2–8 °C until chromatographic analysis.

For the nitrogen atmosphere overlay, the samples were placed in a glove box (Sicco, Grünsfeld, Germany) and gassed with 0.3 mbar nitrogen for 4 h so that the oxygen was displaced to <2.5 hPa (using a Microx 4 trace instrument with a microsensor PM-PSt7 (PreSens GmbH, Germany)). The samples were sealed with Fluro Tec® rubber stoppers (Daikyo, Japan) and then subjected to the same light treatment as mentioned above.

#### 2.1.3 Sample preparation for the protein degradation measurement

Prior to light exposure, the sterile filtered mAb-Ι, ΙΙ and ΙΙΙ formulations were aseptically filled in syringes of glass type 1 (Becton, Dickinson and Company, New Jersey, USA). The fill volume was set to 1.05 ml with air headspace estimated to be 0.1 ml. All mAbs were formulated in 50 mM sodium phosphate buffer, pH 6.2, 159 mM trehalose. Afterwards, the syringes were placed into the photochamber (PharmaEvent, Weiss Technik) horizontally with respect to the light source (Planistar 41–57-SLED-1-VD2-W light sheet) at 25 °C and 60 % relative humidity. The samples were exposed to visible light (λ = 400–800 nm), with an intensity of 8′000 lx (Kaiser et al., 2021) for different time points. Sreedhara et al. (2016) calculated the exposure during production of a protein molecule to be between 43 and 260·10^3^ lx·h in the visible region (Sreedhara et al., 2016). We measured similar light dosage exposures in our facilities (Kaiser et al., 2021). The respective irradiation dose was chosen in consideration of reasonable light conditions during common production and administration processes. Therefore the samples experienced light doses of 0 up to 380·10^3^ lx·h as a worst-case condition. The corresponding dark control samples were stored in light-tight containers and placed in the same photostability chamber (PharmaEvent, Weiss Technik) at 25 °C and 60 % RH. Following light exposure, the samples were stored in light-tight containers at 2–8 °C until further analysis.

#### 2.1.4 Sample preparation for electron paramagnetic resonance (EPR) measurements

Different protein concentrations of mAb-Ι, ΙΙ and ΙΙΙ ranging from 50 to 125 mg·ml^−1^ were filled in 2/4 ml glass type 1 vials (Schott AG, Germany) with a Fluro Tec® rubber stopper (Daikyo, Japan) prior to light exposure. The fill volume was set to 3.6 ml with air headspace estimated to be 0.4 ml (∼ 9 %). The mAbs were formulated in 50 mM sodium phosphate buffer, pH 6.2 with 159 mM trehalose at different protein concentrations. The 4-hydroxy-2,2,6,6-tetramethylpiperidyl-1-oxyl (TEMPOL) concentration was set to 50 μM in solution. The vials were placed into the self-made light container and were exposed to visible light (λ = 400–800 nm) with an intensity of 20′000 lx for variable amounts of time (Blaffert et al., 2018; Haeri et al., 2019).

The light container's LED (light-emitting diode) chain (Reflex LED Strip Set 3 m, Paulmann Licht GmbH, Springe) was wrapped around a beaker and placed at distinct distance of 33 mm from the vials which resulted in a light intensity of 20′000 lx. The temperature was set to 25 ± 2 °C. The corresponding dark control samples were stored in light-tightened aluminum foil and placed in the same photostability chamber with the illuminated sample under otherwise identical conditions. After light exposure, 10–15 μl of the respective solution was filled into a micropipette (BLAUBRAND® intraMARK, Wertheim, Germany) and capped with capillary tube sealant (CRITOSEAL® Leica). The vials were set back into the container until the next sample drawing time point. Dark control samples were again wrapped in aluminum foil. The micropipettes were also wrapped in aluminum foil until further analysis.

### 2.2 Methods

#### 2.2.1 Analysis of oxygen depletion

The oxygen content in the headspace was measured immediately after sample withdraw at individual sampling time points after light exposure. Oxygen concentration profiles upon light exposure were carried out with a needle-type microsensor PM-PSt7 Microx 4 Trace (PreSens GmbH, Germany). Before the oxygen concentration was measured, the sensor was calibrated using a series of premixed gases (between 0 % and 20.88 % O_2_, balanced by N_2_). The concentration of oxygen was measured by inserting the microsensor into the headspace for at least 10 s. The oxygen content in the headspace of the control vial and photoirradiated samples were monitored for the three mAb formulations. The mAb samples were exposed to 0, 240 and 720·10^3^ lx·h (0, 24 and 72 h at 10′000 lx). The relative oxygen content was calculated (Δ oxygen content) by subtracting the respective light-exposed buffer value and the respective dark control value from those of the light-exposed sample at each time point. The so determined relative oxygen content (Δ oxygen difference of light-exposed to dark sample & buffer) was plotted against the cumulative light dosage. The protein integrity (high molecular weight species, monomer and low molecular weight species) was measured with ultra-performance size-exclusion chromatography (UPSEC), as described in the following.

#### 2.2.2 Analysis of protein aggregation by ultra-performance size exclusion chromatography (UPSEC)

Molecular size distribution was determined via ultra-performance size-exclusion chromatography (UPSEC). Therefore, a Waters Acquity UPLC connected with a tunable UV detector (TUV) (Waters, Milford, MA, USA) at 280 nm equipped with an UPLC BEH200SEC 300 x 4.6 mm column (Waters, Milford, MA, USA) was used. The samples were diluted with deionized water to 5 mg·ml^−1^. Each injection contained 30 μg protein. The column was equilibrated and eluted with an isocratic flow of 0.2 ml·min^-1^ with 200 mM L-arginine, 120 mM ammonium sulfate, and 10 % propan-2-ol at pH 7.3. Column elution was monitored for 25 min. Peak integration was performed using the Empower™ software. The peaks integrated include high molecular weight (HMW) species, monomer and low molecular weight (LMW) components. An exemplary chromatogram is shown in the supplementary material section (Fig. S2). Subtracting the respective “dark control” (sample stored in the absence of light exposure) from the sample exposed to light result in the relative change of each time period.

#### 2.2.3 Analysis of hydrophobic structural changes by measuring the hydrophobic interaction chromatography (HIC)

The HIC was used to analyze the hydrophobic structural changes of the mAb. The chromatography was performed on a Waters 2695 Alliance HPLC system connected with a fluorescence detector Waters 2475 (Waters, Milford, MA, USA). The utilized column was a TSKgel® Butyl-NRP column phase C4 10 cm x 4.6 mm for mAb-Ι and -ΙΙ. For mAb-ΙΙΙ a Thermo Scientific MAbPac HIC-10, 5 μm, 4.6 x 250 mm column was applied. The column temperature was set to 40 °C and the autosampler to 8 °C. For mAb-Ι, ΙΙ and ΙΙΙ the mobile phase A contained 1.8 M ammonium sulfate and 0.1 M disodium hydrogen phosphate at pH 7.0 and mobile phase B was 0.1 M disodium hydrogen phosphate at pH 7.0. The elution was achieved by a linear gradient from 10 to 90 % buffer B within 40 min. Subtracting the respective dark control from the light irradiated sample resulted in the relative change at each time period. The prepeaks obtained in the HIC chromatography showed decreased hydrophobicity towards lower HIC retention times in comparison to non-oxidized samples. An exemplary chromatogram is shown in the supplementary material section (Fig. S4).

#### 2.2.4 Analysis of protein oxidation by measuring binding using Protein A chromatography

Analytical Protein A chromatography was used to separate oxidized mAb (modified) isoforms from unmodified mAb proteins due to differences in their affinity towards immobilized Protein A (Gao et al., 2015). Therefore, a Waters 2695 Alliance HPLC system connected with a tunable UV detector (TUV) (Waters, Milford, MA, USA) at 280 nm using a POROS TM A 20 μm 4.6 x 50 mm (Thermo Fisher Scientific, Dreieich, Germany) was used. The column and autosampler temperature were set to 23 °C and 8 °C, respectively. The samples were diluted with Milli-Q to 5 mg·ml^−1^. Each column injection contained 100 μg protein. The Protein A column was equilibrated and eluted using a gradient flow of 2 ml·min^−1^ for mAb-Ι and -ΙΙ. For mAb-ΙΙΙ the mobile phase A contained of phosphate buffered saline (PBS) at pH 7.4 and the mobile phase B 100 mMacetic acid, 150 mM sodium chloride at pH 2.8. The elution was achieved by a linear gradient from 0 to 45 % mobile phase B within 35 min. The relative changes were calculated by subtracting the respective dark control sample from the light irradiated sample. A representative chromatogram is shown in the supplementary material section (Fig. S3).

#### 2.2.5 Analysis of charge variants using ion exchange chromatography (IEC)

IEC resolves different charge variants of mAbs potentially evolving due to multiple posttranslational modifications including those occurring upon photooxidation. These variants can be monitored by weak-cation exchange (WCX) chromatography, where acidic and basic protein variant isoforms can be separated and analyzed. The different isoform variants can be separated according to their overall surface net charge (Trappe et al., 2018). The IEC was performed on a Waters 2695 Alliance HPLC system in combination with a UV/Vis detector Waters 2489 (Waters, Milford, MA, USA). The column was a ProPac WCX-10, 4 x 250 mm (Thermo Fisher Scientific, Dreieich, Germany). The column temperature was set to 30 °C for mAb-Ι, 20 °C for mAb-ΙΙ and 40 °C for mAb-ΙΙΙ. The autosampler temperature was set to 5 °C. An amount of 60 μg was loaded on the column with a flow of 0.8 ml·min^-1^. The elution of the different charged protein variant isoforms was obtained using standard IEC mobile phases as reported in the literature (Du et al., 2012). The peak areas were detected by UV at 280 nm absorbance. The areas were used to calculate the amount of APG (acidic peak group), the main protein isoform and BPG (basic peak group) of each sample. Changes in the relative content of each protein isoforms were calculated by subtracting the respective dark protein containing control sample.

#### 2.2.6 Analysis of protein oxidation by mass spectrometry (MS)

Tryptic peptide mapping with LC-MS/MS was performed to measure the abundance of oxidized Met and Trp residues in mAbs (Nybo et al., 2019). Approximately 700 μg of protein were mixed with 7 M guanidine-HCl to achieve a final protein concentration of 1.0 mg·ml^−1^ followed by a reduction of the disulfide bonds. Therefore, 7 μl of a 1.0 M dithiothreitol (DTT) solution was added and incubated at 57 °C for 20 min at 500 rpm, followed by alkylation with 17.5 μl of 1.0 M iodoacetamide (IAA) at room temperature, stored in the dark, for 20 min. By adding 35 μl of a 1.0 M DTT solution the alkylation reaction was stopped. Remaining denaturing and reducing agents were removed by preparative gravity flow SEC (NAP10 columns). Therefore, the column was prewashed three times with 4 ml of 100 mM ammonium bicarbonate buffer and afterwards the protein solution was added to the column, followed by a washing step after complete infiltration with 400 μl of 100 mM ammonium bicarbonate buffer. The elution was done by pipetting 600 μl of 100 mM ammonium bicarbonate buffer on the column. 300 μL of the eluted sample were subsequently mixed with 300 μL of a 0.1 M methionine solution. The samples were digested by the addition of 12 μl of a 1 mg·ml^−1^ trypsin solution and 6 μL of a 0.1 mg·ml^−1^ RapiGest solution, and subsequent incubation at 37 °C for 30 min at 300 rpm. Digestion reaction was quenched by addition of 5 μL of 100 % formic acid and stored at 2–8 °C for 45 min.

Digested samples were analyzed by LC-MS tryptic mapping using a Waters Acquity UPLC chromatography system coupled to mass spectrometry detection in positive ion mode on a Orbitrap Exploris™ 240 mass spectrometer equipped with a H-ESI ion source (Thermo Scientific, Waltham, MA). About 10 μg protein digest was loaded onto the UPLC system with a Waters XSelect Peptide CSH C18 reverse phase column with the column temperature set to 65 °C at a 0.25 ml·min^-1^ flow rate. The eluents consist of 0.1 % (v/v) formic acid (FA) in deionized water from a MilliQ system (Merck KGaA, Darmstadt, Germany) for mobile phase A, and mobile phase B was 0.1 % formic acid (FA) in LC-MS grade acetonitrile. Peptides were eluted with an isocratic gradient of 0 % B within the first three minutes, followed by a linear gradient of 0–45 % B in 52 min. Afterwards, the column was washed with 90 % B two times for 2 min and then equilibrated with 0 % B for 5 min. MS1 data was acquired in the range of 200–2500 *m*/*z* in full scan mode at a resolution of 250 k. MS2 data was acquired as top 3 experiment in the range of 200–2000 m/z at a resolution of 15 k with HCD collision energies set to 25 % / 30 % / 35 %. Ion source parameters were as follows: spray voltage 3500 V, vaporizer temperature 300 °C, sheath gas ∼5.3 l·min^−1^, auxiliary gas ∼8.3 l·min^−1^, and ion transfer tube temperature 350 °C. Raw data was evaluated by Byos software version 4.2–370 using nodes Byonic and Byologic from Protein Metrics Inc. (Cupertino, CA). Wildtype and modified peptides were accepted for quantification in case their measured accurate molecular mass and isotopic distribution were in good agreement with the theoretical molecular mass and isotopic distribution. In addition, they were identified by MS2 data. The relative quantities of oxidized peptides containing Met (methionine) or Trp (tryptophane) were calculated from dividing extracted ion chromatogram peak areas of the sum of the wildtype and the modified peptide by the modified peptide. The precision of the relative quantification of oxidation levels is ±0.2 % to ±0.5 %, depending on the peptide. The mAb were numbered accordingly to the primary sequence.

#### 2.2.7 Electron paramagnetic resonance (EPR) measurement

Electron paramagnetic resonance spectroscopy was performed to measure the radical formation upon visible light exposure. All continuous wave EPR spectra were measured using the Miniscope MS 5000 (Magnettech GmbH, Berlin, and Freiberg Instruments, Freiberg, Germany) and controlled by the Freiberg Instruments software. The temperature was controlled using a MS 5000 (Magnettech GmbH, Berlin, Germany). Micropipettes (BLAUBRAND® intraMARK, Wertheim, Germany) were filled with about 10–15 μL of sample solution containing 50 µM TEMPOL and capped with capillary tube sealant (CRITOSEAL® Leica) and placed into the spectrometer. The temperature was set to 25 °C with ±0.2 °C. For all X-band measurements a magnetic field sweep of 8 mT centered around 337.6 mT with a scan time of 60 s, a modulation of 0.05 mT (100 kHz) and a microwave power of 5 mW were used. Each spectrum is an accumulation of 5 scans. The raw EPR spectra (first derivative) were integrated to obtain the microwave absorption spectra, which were baseline corrected. After that, the center field peaks were integrated to obtain the double integrals (DI).

## 3 Results and discussion

The experimental setup for mAb photodegradation and radical formation were characterized with respect to the exposed light dose. These parameters can be compared with conditions encountered in development laboratories, manufacturing facilities and administration processes. All measurements were carried out using visible light. Previously we have shown that the cumulative light dosage of visible light can affect product quality of IgG mAbs (Kaiser et al., 2021). Following exposure to visible light, the mAb formulations were analyzed for headspace oxygen content, physical and chemical protein degradation, and radical content.

### 3.1 Impact of visible light on the headspace oxygen content

**Fig. 1:**
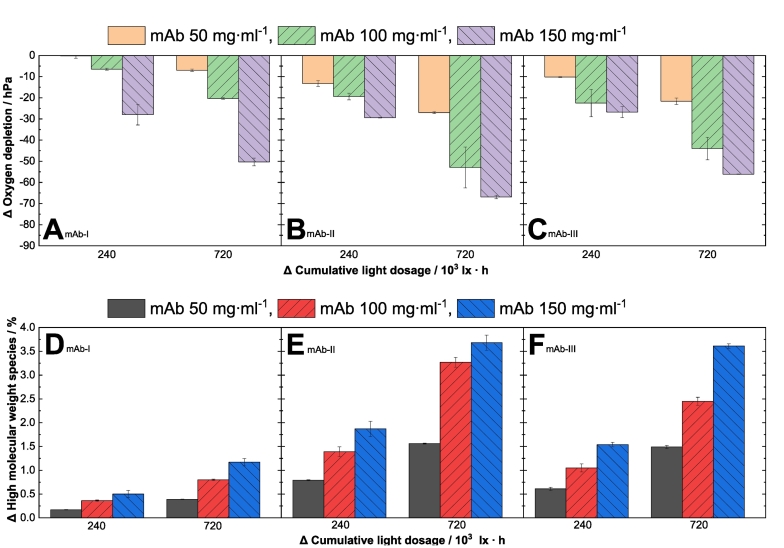
(A, B, C) Photoinduced oxygen content reduction of mAb-Ι, ΙΙ and ΙΙΙ. Oxygen content measurement in the headspace of vials for three monoclonal antibody formulations in phosphate buffer at pH 6.2, V = 3.9 ml with three protein concentrations ( 50 mg·ml^−1^,  100 mg·ml^−1^,  150 mg·ml^−1^). The relative oxygen depletion is depicted on the y-axes (light-exposed sample subtracted from dark control and buffer sample), the relative cumulative light dosage is depicted on the x-axes at 240 and 720 ·10^3^ lx·h. The dark protein containing samples and buffer control were used to exclude impact of temperature and buffer components. (D, E, F) Photoinduced formation of HMW of different mAbs. Changes in the relative content of high molecular weight species were measured by size-exclusion chromatography. The mAb-Ι, ΙΙ and ΙΙΙ series, at different protein concentrations ( 50 mg·ml^−1^,  100 mg·ml^−1^,  150 mg·ml^−1^) in phosphate buffer at pH 6.2 were exposed to visible light with at 0, 240·10^3^ to 720·10^3^ lx·h in glass vials. HMW species are plotted against the cumulative light dosage. All investigated samples were free of polysorbate.

We investigated the effect of visible light on the oxygen content in the headspace of vials in three different mAb formulations. The different antibodies at protein concentrations of 50, 100 and 150 mg·ml^−1^ were filled in 2/4 ml glass type 1 vials with 3.90 ml. The headspace volume was estimated to be 0.10 ml. The so prepared protein samples were exposed to two different light dosages. The effect of visible light on the oxygen content in the headspace of mAb solutions is shown in Fig. 1, revealing a correlation between the cumulative light dose and the decrease of the oxygen content in the headspace. For example, for 150 mg·ml^−1^ mAb-Ι, an increase in the cumulative light dosage from 240·10^3^ to 720·10^3^ lx·h results in a higher increase in the change of relative oxygen content, and thus in a decrease in total oxygen content from −28 hPa (−38 μmol·l^-1^) to −50 hPa (−68 μmol·l^-1^), respectively (see Fig. 1A). This correlation is found in all tested mAb formulations (Fig. 1). None of the samples stored in the dark showed a change in oxygen depletion (supplementary material section, Fig. S5). The headspace oxygen content decreased, leading to protein oxidation (see further data Fig. 1D–F). These results suggest that an unknown photosensitizer absorbs and transfers energy or electrons to dissolved oxygen molecules, yielding oxygenated radical species and/or singlet oxygen that oxidize the present protein (Schöneich, 2020). These oxygenated radicals can then chemically interact with the proteins, leading to a decreased oxygen content in the solution.

**Fig. 2:**
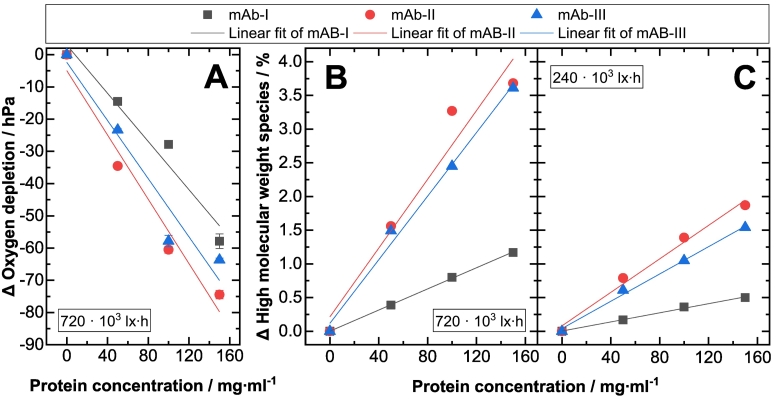
Correlation of the three tested mAbs I-III ( mAb-Ι,  mAb-ΙΙ,  mAb-ΙΙΙ) at different protein concentrations with (A) the change (Δ) in oxygen depletion and (B, C) the change in the formation of high molecular weight species (ΔHMW). The three monoclonal antibody formulations are buffered in phosphate at pH 6.2, V = 3.9 ml. The investigated samples were free of polysorbate. (A) Correlation of protein concentration and oxygen depletion of three mAbs at 720·10^3^ lx·h cumulative light dosage measured in glass vials. The linear fit (supplementary material section, Table S1) describes the relationship between the reduced oxygen content and the protein concentration upon light exposure. (B, C) Correlation of protein concentration and the change (Δ) in the formation of the high molecular weight species of three mAbs at 720·10^3^ (B) and 240·10^3^ (C) lx·h cumulative light dosage measured in glass vials. The linear fit (supplementary material section, Tables S2 and S3) describes the relationship between the high molecular weight species and mAb concentration upon light exposure.

As there is an equilibrium between dissolved oxygen in solution (8–9 mg·dm^−3^ (Mortimer, 2017)) and in the headspace (gas phase) of the vials, the initial reactions yielding to oxygenated radicals and/or singlet oxygen in solution lead to a decreased oxygen content in the headspace. In addition, keeping the cumulative light dosage constant, the decrease in headspace oxygen content is correlated with the protein concentration (Fig. 2A). For example, in solutions of mAb-Ι exposed to 720·10^3^ lx·h, a decrease of −5 hPa (−7 μmol·l^-1^) for 50 mg·ml^−1^, −20 hPa (−27 μmol·l^-1^) for 100 mg·ml^−1^ and -50 hPa (−68 μmol·l^-1^) for 150 mg·ml^−1^ protein concentration was observed, respectively (Fig. 1A). This correlation between protein concentration and oxygen depletion in the headspace was observed for all investigated mAb proteins (Fig. 1) indicating a higher concentration of the photosensitizer with higher protein concentrations. However, the individual sensitivity of the mAb proteins to the oxygen content decrease was different (Fig. 1). The largest differences between the dark and light-exposed samples were detected in the case of mAb-ΙΙ (−70 hPa) (Fig. 1B).

The oxidation of proteins depends on intrinsic factors, (i) of primary sequence and (ii) the overall structure and flexibility and dynamics that reside in the carbon-alpha backbone of the polypeptide chain including amino acid side chains with more or less degree of freedom for internal rotation (rotamers). In addition to intrinsic factors, extrinsic factors such as solution pH, ionic strength, buffer composition and excipients may affect photooxidation of proteins. “Extrinsic” factors in our case were kept constant, i.e. same excipient batch used for all formulations. Therefore, one may conclude that intrinsic factors lead to a different mAb sensitivity towards photodegradation. The oxidation of the proteins may vary due to different primary sequences (Marinelli et al., 2018) and conformational stability and flexibility (Saphire et al., 2002), influencing the accessibility of amino acid residues (Bommana et al., 2018) against reactions of ROS species and formulation composition (Prajapati et al., 2022; Shah et al., 2018). Additionally, amino acids such as Met and Trp facing the solvent are more susceptible to oxidation than buried residues in the hydrophobic core of the protein (Hensel et al., 2011; Wei et al., 2007; Folzer et al., 2015). Using our current setup, for the detection of a significant change in the oxygen content, a small headspace volume needs to be chosen in relation to a large volume of protein solution (1.25 %).

### 3.2 Effect of visible light on the formation of high molecular weight (HMW) species for different antibody formulations

The implications of the oxygen consumption data presented above were further explored by measuring the degradation profiles of the mAbs as a function of their concentrations. Using size exclusion chromatography (SEC), we studied the changes in the low molecular weight species (LMW) and higher molecular weight (HMW) content of the three mAb formulations. In accordance with literature (Sreedhara et al., 2016; Luis et al., 2018; Kaiser et al., 2021) the SEC results show increasing HMW formation upon visible light exposure, while fragmentation is not significantly increased (supplementary material section, Table S5). None of the dark control samples show the formation of HMW species in comparison to the initial samples (supplementary material section, Table S5). As described in the literature, the relative amount of HMW species increases with cumulative light dosage, for example for mAb-Ι 150 mg·ml^−1^ the relative HMW content rises from 0.5 % to 1.2 % when increasing the light dosage from 240·10^3^ to 720·10^3^ lx·h (Fig. 1D–F) (Kaiser et al., 2021; Qi et al., 2009). Furthermore, an enhanced formation of ΔHMW is observed with increasing protein concentration. MAb-Ι features relative amounts in ΔHMW species that increase from 0.4 % to 0.8 % and 1.2 % at concentrations of 50 mg·ml^−1^, 100 mg·ml^−1^, and 150 mg·ml^−1^, respectively at a light exposure of 720⋅10^3^ lx⋅h. For mAb-II and mAb-III formulated at 150 mg⋅ml^−1^ and exposed to 720⋅10^3^ lx⋅h, an ΔHMW increase up to 3.5 % is observed (Fig. 2B). This trend in the concentration dependency was observed for all three mAbs investigated in this study (Fig. 2B, C). However, the change of HMW formation differs between the three monoclonal antibodies. The propensity to form HMW at identical concentrations decreases in the order of mAb-ΙΙ > mAb- ΙΙΙ > mAb-Ι (Fig. 2), which is the same order described for the oxygen content measurement above. The protein concentration shows a direct linear correlation to the HMW content (Fig. 2B, C) (supplementary material section, Table S2, S3). The difference in HMW content of the three mAb formulations can be explained by protein specific variations in amino acid sequence (Marinelli et al., 2018) and conformational instabilities (Saphire et al., 2002), which can influence the accessibility of ROS to specific amino acids (not in focus of this study). Especially surface-exposed amino acids, which are also prone to oxidation can influence the conformational stability of proteins (Hensel et al., 2011; Thirumangalathu et al., 2007), thereby leading to a higher tendency for self-association and decreased stability (Semagoto et al., 2014). Further information of the aggregation process upon photooxidation is given in Kaiser et al. (2021) (Kaiser et al., 2021). Furthermore, the absorption spectrum of a potential protein-bound photosensitizer (unknown yet) can be influenced by conformational flexibility and dynamics, as Spezia et al. (2003) showed (Spezia et al., 2003). As highlighted by Mantha et al. (2020), the presence of specific compounds such as riboflavin or ascorbic acid, that may act as photosensitizer, impairs the photostability of protein formulations, via the formation of reactive oxygen species that oxidizes the present proteins (Mantha et al., 2020). Even advanced glycations were reported by the authors (Mantha et al., 2020). Although, they investigated protein photostability using an in vitro vitreal assay, similar degradations may be encountered during long term drug product storage. Due to such observations, Du et al. (2018) discussed some mitigation options, such as an adequate light source to avoid or reduce photodegradation of protein (Du et al., 2018).

**Fig. 3:**
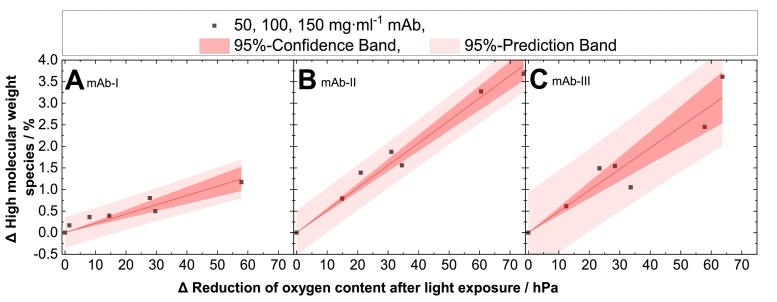
Correlation between decreased oxygen content and HMW of three mAbs. Photoinduced protein particle formation as measured by SEC, is plotted against the reduced oxygen content for (A) mAb-Ι, (B) mAb-ΙΙ and (C) mAb-ΙΙΙ. Relative changes (Δ light exposed sample subtracted from the protein sample stored in the “dark”) of the concentration-independent formation of high molecular weight species were determined and plotted against the relative reduction of the oxygen content measured (Δ light exposed samples subtracted from the protein containing sample and the buffer sample stored in the “dark”) in glass vials. The linear fit (supplementary material section, Table S4) describes the relationship between the reduced oxygen content and the HMW upon light exposure.

**Fig. 4:**
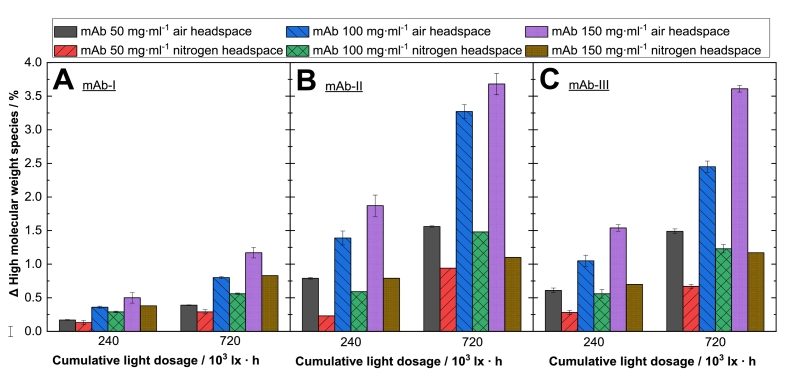
Photoinduced high molecular weight formation (HMW) (Δ light exposed sample subtracted from protein containing sample stored in the “dark”) of different mAbs under nitrogen and air atmosphere headspace in glass vials. The (A) mAb-Ι, (B) mAb-ΙΙ and (C) mAb-ΙΙΙ in phosphate buffer at pH 6.2 were exposed to visible light with 0, 240·10^3^ and 720·10^3^ lx·h. Changes in the relative content of HMW species were measured by SEC and plotted against the cumulative light dosage. Three monoclonal antibody concentrations are marked in different colors and symbols with either air or nitrogen headspace ( 50 mg·ml^−1^: air,  50 mg·ml^−1^: nitrogen,  100 mg·ml^−1^: air,  100 mg·ml^−1^: nitrogen,  150 mg·ml^−1^: air,  150 mg·ml^−1^: nitrogen). The relative changes of high molecular weight species (Δ HMW) were obtained by subtracting the light exposed protein containing samples from the samples stored in the “dark”. The investigated samples were free of polysorbate.

Considering the results reported in Fig. 2A–C, the reduction (as compared to the dark reference) in the oxygen content of light-exposed protein samples and the formation of HMW species as a function of protein concentrations shows, that oxygen depletion and HMW increase, scale linearly with protein content (Fig. 2A–C) (supplementary material section, Table S3). Since the oxygen depletion correlates linearly with the HMW formation, a decrease of oxygen is an indicator for protein degradation (Fig. 3A–C). The correlation coefficient (R^2^) is 0.94 for mAb-Ι, 0.99 for mAb-ΙΙ and 0.96 for mAb-ΙΙΙ (supplementary material section, Table S4). The relative oxygen depletion was positively correlated with the protein HMW species. A higher oxygen content decrease corresponds to a stronger protein particle formation upon light treatment. Tan et al. (2021) found a positive correlation between fill level and degree of oxidation under the influence of light for milk proteins (Tan et al., 2021). Our results so far indicate that headspace and dissolved oxygen have an effect on the oxidative reactivity of mAbs in aqueous solution (Fig. 4). Minimizing the oxygen content to 2.5 hPa instead of ∼220 hPa by overlaying inert gas such as nitrogen in the headspace leads to a decreased protein oxidation of up to 70 %, as shown in Fig. 4 for the tested systems. This is especially obvious for mAb-II and mAb-III, where for the 150 mg⋅ml^−1^ formulations exposed to 720⋅10^3^ lx⋅h, a HMW reduction from approx. 3.5 % to approx. 1 % HMW is achieved by replacing the air headspace by nitrogen (Fig. 4). For mAb-Ι the oxidation under nitrogen decreased up to 30 %, for mAb-ΙΙ up to 70 % and for mAb-ΙΙΙ around 70 % (Fig. 4). These results indicate that protection of protein formulations from photodegradation can be achieved by minimization of the oxygen content headspace of the primary container or the use of inert gas instead of air (Qi et al., 2009). Thus, reducing the oxygen content in the headspace and using an impermeable oxygen packaging material may lead to a less pronounced photooxidation for light sensitive proteins (Gupta et al., 2021; Masato et al., 2015; Won et al., 2020; Härdter et al., 2021; Landi and Held, 1986).

### 3.3 Effect of photooxidation on protein stability considering the protein concentration

As depicted above, the protein concentration has an influence on the photostability considering the oxygen depletion and the related HMW formation of proteins. Confirming the obtained results, we focused on protein degradation using different chromatographic methods as well as mass spectrometry. Different protein concentrations were investigated. The headspace was set larger than 2.5 % of the total volume (in comparison to chapter before using vials, ∼9 % headspace) and filled into glass syringes. Size-exclusion chromatography was performed to monitor degradation upon light exposure. Additionally, we studied whether the formation of HMWs is changed in dependence of the protein concentration upon visible light exposure and the presence of PS20 HP (high purity), whereas type and concentration of excipients play a minor role in this study. Therefore, we investigated three mAbs with concentrations of 5, 10, 20 and 90 mg·ml^−1^ in the absence and in the presence of PS20 HP. One formulation contained PS20 HP as additional excipient at 0.4 mg·ml^−1^, a concentration usually used to stabilize mAb biologics (Martos et al., 2017). As a positive control, another sample was formulated with a ten times higher PS20 HP concentration of 4.0 mg·ml^−1^ to intensify the observed effect. More information of the PS20 HP containing samples is given in the next section. Our results show an increased formation of protein HMWs with higher protein concentration and the presence of PS20 HP.

**Fig. 5:**
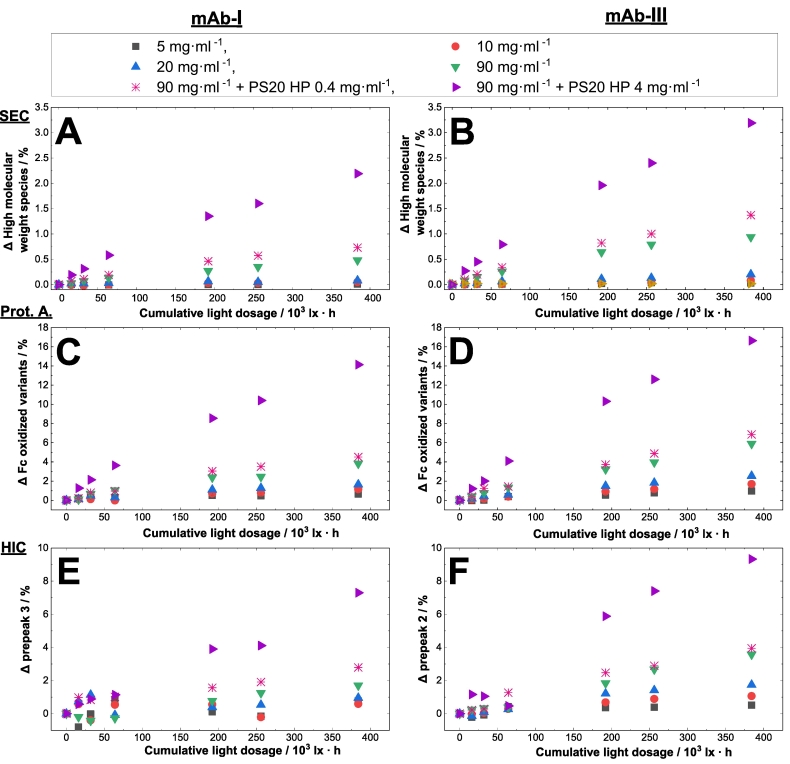
Photoinduced protein physical and chemical changes upon visible light exposure was exemplary shown for mAb-Ι and mAb-ΙΙΙ. The mAb-Ι and ΙΙΙ formulation is in phosphate buffer at pH 6.2. Different monoclonal antibody concentrations with and without different polysorbate 20 HP concentrations in glass syringes are marked with different colors and symbols ( 5 mg·ml^−1^,  10 mg·ml^−1^,  20 mg·ml^−1^,  90 mg·ml^−1^,  90 mg·ml^−1^ + 0.4 mg·ml^−1^ polysorbate 20 HP,  90 mg·ml^−1^ + 4 mg·ml^−1^ polysorbate 20 HP). (A, B): Photoinduced HMW formation of mAb-Ι and ΙΙΙ. Changes (Δ) in the relative content of HMWs was measured for mAb-Ι and ΙΙΙ upon exposure to visible light. The cumulative light dosage is plotted against the high molecular weight species. (C, D): Photoinduced Fc oxidation variants of mAb-Ι and ΙΙΙ. Changes (Δ) in the relative content of Fc oxidized variants (measured by Protein A affinity chromatography) of mAb-Ι & ΙΙΙ are shown after exposure to visible light. The cumulative light dosage is plotted against the Fc oxidized variants. (E, F): Photoinduced hydrophobicity change of mAb-Ι and ΙΙΙ. Changes (Δ) in the relative content of the prepeaks (HIC) of mAb-Ι & ΙΙΙ are shown after exposure to visible light. The cumulative light dosage is plotted against the change in the prepeak obtained from HIC.

**Fig. 6:**
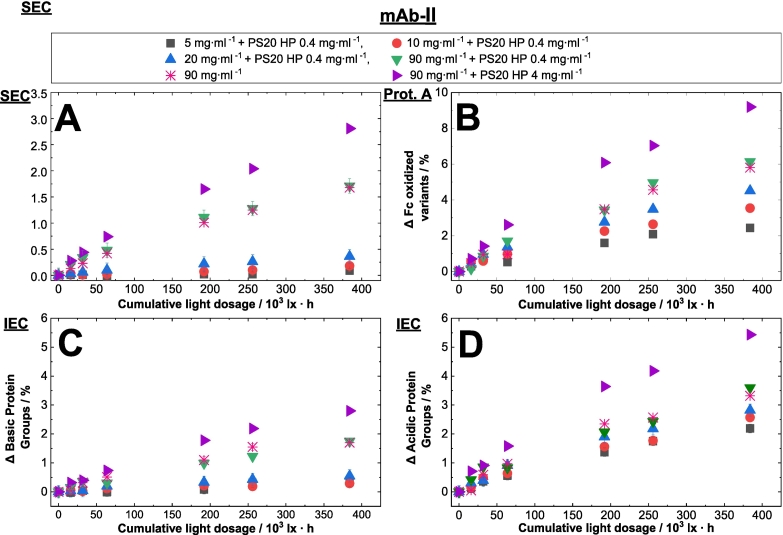
Detection of various photooxidized protein species via different chromatographic techniques. For each chromatographic technique, including SEC (A), analytical Protein A (B) and IEC (C and D) the protein concentration dependence upon light treatment is shown. Changes (Δ) in the relative content of HMW (A), Fc oxidized protein variants (B), APG (C) and BPG (D) of mAb-ΙΙ in phosphate buffer pH 6.2 are shown after exposure to visible light treated in glass syringes. The cumulative light dosage is plotted against the change in the chromatographic peaks. Different monoclonal antibody concentrations in glass syringes in the absence and presence of different polysorbate concentrations are marked in different colors and symbols ( 5 mg·ml^−1^ + 0.4 mg·ml^−1^ polysorbate 20 HP,  10 mg·ml^−1^ + 0.4 mg·ml^−1^ polysorbate 20 HP,  20 mg·ml^−1^ + 0.4 mg·ml^−1^ polysorbate 20 HP,  90 mg·ml^−1^ + 0.4 mg·ml^−1^ polysorbate 20 HP,  90 mg·ml^−1^,  90 mg·ml^−1^ + 4 mg·ml^−1^ polysorbate 20 HP).

The degradation with increased protein concentration was investigated for three mAb formulations containing different protein concentrations (Fig. 5). For example, for mAb-Ι, the HMW value increased about 5-fold from 0.1 % to 0.5 % when the concentration was raised from 5 to 90 mg·ml^−1^ (exemplary chromatogram for SEC see supplementary material section, Fig. S2). In this regard, an increase in HMW was seen for all detected samples (Fig. 5A, B). Protein oxidation can occur at various amino acid residues such as tryptophan (Trp), tyrosine (Tyr), and histidine (His), however, methionine (Met) oxidation has been reported to be most likely (Levine et al., 2000). The Met residues in the Fc region (Met252 and Met428; Kabat convention) are well-known hot-spots of light-induced chemical changes in mAbs (Loew et al., 2012). Changes in the conformation of the Fc region, such as methionine oxidation at the CH_2_ and CH_3_ domains can be identified by Protein A chromatography (Pan et al., 2009). Oxidation of these Met residues can lead to a reduction of the Fc- gamma receptor binding affinity (Pan et al., 2009). The distance between the sulfur atom in Met252 and atoms in Protein A such as Phe124 is of <4 Å, so that methionine oxidation to sulfoxide is very likely to disturb the hydrophobic interaction (Loew et al., 2012; Pan et al., 2009). Changes in the Fc domain such as oxidation of M252 and M428 weaken the binding of the intact antibody to Protein A, which results in slightly higher eluting pH conditions of the oxidized variant (Pan et al., 2009) (an exemplary Protein A chromatogram is shown in the supplementary material section, Fig. S3). Exposure up to 384·10^3^ lx·h of 5 mg·ml^-1^ of mAb-Ι results in a relative increase in Fc-oxidized variants of 0.7 %, while at 90 mg·ml^−1^ of mAb-Ι led to a relative increase of Fc variants of 3.8 % (see Fig. 5C). The same protein concentration dependence can be seen for mAb-ΙΙ and -ΙΙΙ formulations (see Fig. 6B for mAb-ΙΙ and Fig. 5D for mAb-ΙΙΙ). Protein species can be separated rapidly without denaturation by hydrophobic interaction chromatography. In mAb-Ι formulation, an increase in the protein concentration from 5 mg·ml^-1^ to 90 mg·ml^−1^ leads to a two-fold increase in prepeak area from 0.8 % to 1.7 % (see Fig. 5E) (An exemplary HIC chromatogram is shown in the supplementary material section, Fig. S4). Changes in the prepeak of mAb-Ι and mAb-ΙΙΙ could be investigated using HIC, for mAb-ΙΙ changes in the IEC peak profile was used. IEC peak profile can be attributed to the alterations in surface charge distribution on the protein because of oxidation in Met, Trp and other residues. Even small changes in the mAb structure may change the local distribution of charged residues, leading to alterations in the overall surface charge distribution of the protein molecule (Vlasak and Ionescu, 2008). Here for mAb-ΙΙ, cation-exchange chromatography was used, with acidic protein species (acidic peak groups, APG) variants eluting prior to the main peak and basic protein species (basic protein group, BPG) (Du et al., 2012). As depicted in Fig. 6, the higher the protein concentration, the higher the APG and BPG species upon light incubation for mAb-ΙΙ. For mAb-Ι and mAb-ΙΙΙ no difference between light-exposed and dark samples were seen for IEC (data not shown).

LC-MS was performed to identify the molecular changes of Trp and Met oxidation. Different Met and Trp residues are prone to oxidation to different extents depending on the location in the protein's polypeptide chain and its surface exposure to the solvent. As mentioned above, prone Met residues are typically located on the surface of protein residues and show correlation with their individual flexibility in context to their allocation within the polypeptide chain and their surrounding/neighboring residues (Chu et al., 2004; Hui et al., 2015). Therefore, we measured the Met and Trp oxidation as shown in Table 1 and Table 3. Exemplary shown for mAb-Ι, Met and Trp oxidation increases with higher protein concentrations (Table 1). Met251 (mAb-Ι) is significantly increased at 20 mg·ml^−1^ and at 90 mg·ml^−1^. Met357 (mAb-Ι) does not show significant changes in oxidation as it is buried in the surface. The LC-MS data from Trp416/ Met427 (mAb-Ι) are confirming the results from the Protein A chromatography, which show an increased amount of Met and Trp oxidation with increasing protein concentration (for Protein A chromatography a loss in Fc binding affinity). Trp416 and Met427 are on the same peptide, and therefore cannot be considered separately.

**Table 1:** Proteolysis-coupled mass spectrometry data of relevant mAb-Ι photooxidized Met and Trp residues.

Concentration of mAb-Ι	Condition	Light dosage	Met 251	Met357	Trp416 & Met427
/ mg·ml^−1^		/ 10^3^ lx·h	/ %	/ %	/ %
90	initial	0	4.0	0.6	1.8
90	dark	0	4.1	0.6	1.8
5	light	384	4.5	0.8	2.2
10	light	384	4.1	0.7	1.9
20	light	384	4.9	0.7	2.3
90	light	384	5.9	0.8	2.9

Based on the SEC, Protein A, IEC, HIC chromatography and LC-MS results for the investigated mAbs, the impact of visible light on product stability depends on the protein concentration. The propensity to form HMW, oxidized variants measured by Protein A chromatography for mAb at identical protein concentrations decreases in the order of mAb-ΙΙ > mAb-ΙΙΙ > mAb-Ι, which corresponds to the same order described for the oxygen content measurement studied above. The authors Mason et al. (2012) described a similar protein-concentration dependent photooxidation upon UV exposure (Mason et al., 2012). However, since proteinogenic structures absorb light solely in the UV range, the increase of HMWs with increasing protein concentration may be related to the intrinsic absorption of proteinogenic structures. In contrast, our results were obtained upon visible light exposure without intrinsic absorption from the protein itself, since proteinogenic structures absorb light up to 360 nm (e.g Kynurenine), but not into the visible range (up to 400 nm) (Schöneich, 2020).

Distinct differences were seen in the chromatographic measurements upon light exposure of mAbs at different protein concentrations. For the initial time points for all concentrations, in a first approximation, a “linear” increase in HMW, Fc-oxidized variants and acidic species is observed, followed by an increase with a smaller slope over the remaining light exposure time. Mason et al. (2012) described the shallowing increase of HMW upon UV light exposed samples Mason et al. (2012) without explaining the trend. Without providing a decent explanation, our assumption of a first-order like kinetic could be due to an unstable photocatalyst, which might decompose to another more inefficient one, that slow down the reaction kinetic. Radical species may inhibit the chain reaction by reacting with the chromophore of the photosensitizer. A decreased substrate oxygen content can be excluded as a potential reason, as the headspace volumes are large for most commercial setups (>2.5 % compared to the fill volume, here ∼9 %). Gaseous oxygen can diffuse into the solution and may form radical species upon photoreaction (*D*_*O*_2__= 2·10^−5^ cm^2^·s^-1^ at 20 °C (Xing et al., 2014)). Therefore, a change in the chromophore of the photosensitizer may take place. The conversion to another more inefficient photosensitizer is also seen by EPR measurement (following chapter) showing first-order derivative radical residues damaging the chromophore, which might lead to a decreased formation of radicals or singlet oxygen. This in turn leads to less oxidation of the proteins, which was observed in this study.

### 3.4 Polysorbate influence on photooxidation stability

In order to investigate protein stability in the presence of PS20 HP, a visible light study in the presence of two different PS20 HP concentrations were investigated (0.4 mg·ml^−1^ and 4.0  mg·ml^−1^ PS20 HP).

Fig. 5, Fig. 6 show the HMW and Fc oxidation variants of the mAbs in presence of PS20 HP at concentrations of 0.4 and 4.0 mg·ml^−1^, respectively. The presence of PS20 HP resulted in an increase in HMW species of the mAb formulations upon light exposure in comparison to light exposed samples without PS20. Neither an increase in the HMW content nor in the Fc oxidation variants were observed in the dark control samples (data not shown). Without PS20 HP, an increase of 0.4% of HMW can be seen upon light treatment of 384·10^3^ lx·h for mAb-I in comparison to the dark protein sample. With 0.4 mg·ml^−1^ PS20 HP in 90 mg·ml^−1^ mAb-Ι formulations, an increase of 1.5-fold to 0.7 % can be seen (see Fig. 5A). Similar effects are observed for mAb-III (Fig. 5B). In the presence of 4 mg·ml^−1^ PS20 HP, an even stronger increase of 4.5-fold in HMW content to 2.2 % is observed after subjecting the sample to 384·10^3^ lx·h. The chromatographic and LC-MS analysis (Table 2, Table 3) confirm that the higher the PS20 HP content in the mAb formulation, the higher the HMW content upon light exposure. The increased levels of HMW and Fc oxidized variants with increased PS20 HP concentrations may be related to the autooxidation reaction upon light exposure (Kerwin, 2008). These reactions can lead to radical-induced reactions such as oxidation of Trp in mAbs with PS20 (Lam et al., 2011) whereby protein oxidation may lead to the formation of HMWs. The results suggest that byproducts from PS20 introduced through the presence of the surfactant might absorb light in the visible range and act as a photosensitizer. Although the main structure of PS20 HP itself possess no chromophore in the visible range, the absorbance in this spectrum remains unclear (Wuelfing et al., 2006). PS20 HP is a heterogenous mixture (Martos et al., 2017; Zhang et al., 2015; Ayorinde et al., 2000), which composition arises from the polydispersity of the PEG (polyethylene glycol) unit (Tani et al., 1997), the degree of esterification, the uncontrolled dehydration of the sorbitol and fatty acid composition (Ravuri, 2018). In addition, homolytic cleavage of remaining peroxides is partly described as an initiator in oxidation (Ha et al., 2002). However, the homolytic cleavage is only described in the UV range (up to 260 nm) and not in the visible light range (Mierzwa et al., 2018). PS20 is available at different quality grades (Singh et al., 2012), thus having a different impurity profile caused by the synthesis and manufacturing process of the surfactant. The HP grade has a yellowish color, therefore impurities or byproducts from manufacturing could be responsible for absorbing substances in the heterogeneous mixture of PS20 HP solutions. Furthermore, Kishore et al. (2011) compared an initial PS20 solution and PS20 solution stored for 6 months (40 °C), where they observed a peak shift into the visible range (Kishore et al., 2011), which goes along with the yellowish color of PS20 solutions. On the other hand, it is also possible that the interaction between PS20 and protein contributes to higher oxidation rates by enhancing accessibility to the solvent (Prajapati et al., 2020; Lei et al., 2021).

**Table 2:** Proteolysis-coupled mass spectrometry data of relevant mAb-Ι photooxidized Met and Trp residues.

Concentration of mAb-Ι	PS20 HP	Condition	Light dosage	Met251	Met357	Trp416 & Met427
/ mg·ml^−1^	/ mg·ml^−1^		/ 10^3^ lx·h	/ %	/ %	/ %
90	0	Initial	0	4.0	0.6	1.8
90	0	Dark	0	4.1	0.6	1.8
90	4.0	Dark	0	4.4	0.6	1.9
90	0	Light	384	5.9	0.8	2.9
90	0.4	Light	384	6.1	0.7	3.3
90	4.0	Light	384	11.5	1.5	6.9

**Table 3:** Proteolysis-coupled mass spectrometry data of relevant photooxidized Met and Trp residues of mAb-ΙΙ and in the presence PS20 HP.

Concentration of mAb-ΙΙ	PS20 HP	Condition	Light dosage	Met258	Met364	Trp423 & Met434
/ mg·ml^−1^	/ mg·ml^−1^		/ 10^3^ lx·h	/ %	/ %	/ %
90	0	Light	384	11.9	2.1	3.9
90	4.0	Dark	0	6.9	1.4	2.1
90	4.0	Light	384	16.1	2.6	5.8

### 3.5 Protein concentration affects the formation of radicals measured by electron spin resonance (EPR) spectroscopy

The principle of the detection of reactive free radical formation (ROS and carbon-centered radicals) using TEMPOL is based on monitoring the decrease in its EPR intensity (Victória et al., 2022) due to the interaction of the nitroxide radical with the generated ROS species and singlet oxygen in the sample (Victória et al., 2022; Matsumoto et al., 2009; Davies, 2016; Mittag et al., 2022). The formed species may be singlet oxygen (^1^O_2_), superoxide anion (O_2_^−^•), hydroxyl radicals (OH•) or different organic radicals (R•). All these species combined will be termed as “reactive species”, because the reaction with nitroxide radicals will cause a loss of paramagnetism by one electron reduction or one electron oxidation resulting in the formation of hydroxylamines and oxo-ammonium cations, respectively (Hipper et al., 2021; Polovka et al., 2003).

The photogeneration of reactive free radical intermediates in mAb formulations upon visible light exposure was studied by monitoring the intensity of the TEMPOL EPR spectrum upon irradiation of the samples with different mAb concentrations. The photostability of nitroxide free radical TEMPOL under the given experimental conditions (temperature and light exposed buffer control) was high (>0.96 % after 340·10^3^ lx·h, see supplementary material section, Fig. S.6), since only a negligible reduction of its concentration was observed during photoirradiation of mAb-free (buffer) formulations. The magnitude of this effect can be assessed quantitatively by double integration (DI) of the modulated (i.e. kind of first-order derivative) spectra. The double integral (DI) was calculated from each EPR spectrum and then normalized to the DI before light exposure (DI_0_). Visible light irradiation of TEMPOL buffer formulation without mAb formulation did not affect the EPR signal intensity of the spin probe (supplementary material section, Fig. S6).

### 3.6 Effect of visible light on TEMPOL concentration in mAb formulations

**Fig. 7:**
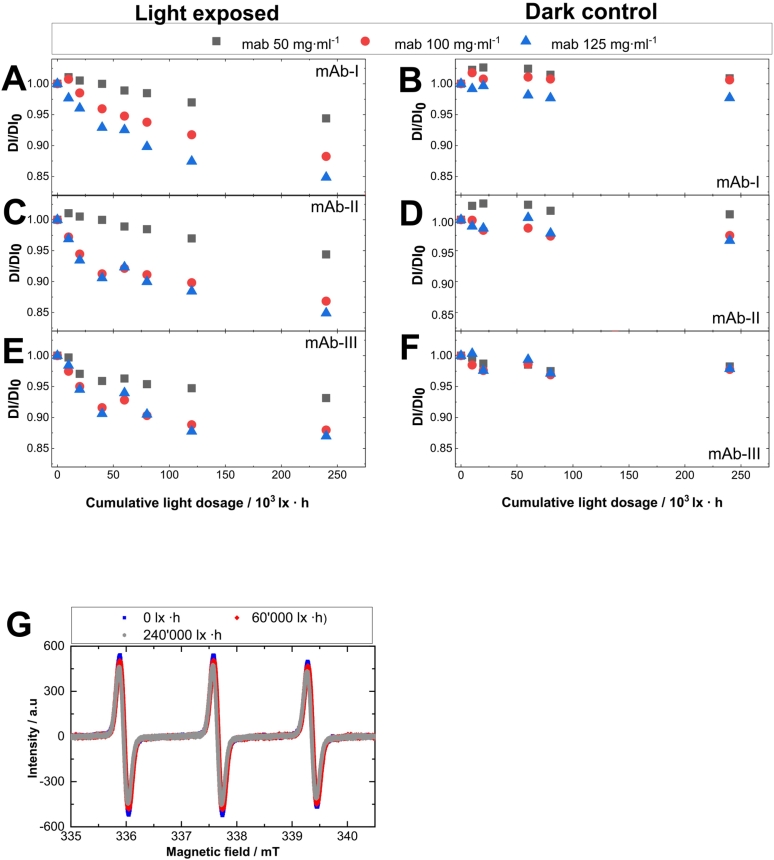
The relative integral TEMPOL intensity evaluated from experimental EPR spectra measured up to 240·10^3^ lx·h light exposure of three different mAb formulations. The double integral (DI) was calculated from each EPR spectrum and then normalized to the DI before light exposure (DI_0_). Decrease of 50 μM TEMPOL in mAb-Ι, ΙΙ and ΙΙΙ formulations in phosphate buffer at pH 6.2 during exposure with broad spectrum of visible light (A, C, E) and protein containing samples stored in the dark (dark control) (B, D, F). Different monoclonal antibody concentrations are marked with different colors and symbols ( 50 mg·ml^−1^,  100 mg·ml^−1^,  125 mg·ml^−1^). The investigated samples were free of polysorbate. (G) Exemplary decrease of EPR TEMPOL signal containing mAb- ΙΙ at 125 mg·ml^−1^ formulation exposed to different cumulative light dosages up to 240·10^3^ lx·h.

Fig. 7 shows the decrease of the TEMPOL EPR signal during exposure to a broad spectrum of visible light between 400 and 800 nm. After 10, 20, 60, 80 and 240·10^3^ lx·h EPR spectra of each mAb-solution were measured. The results of the exposed samples showed a non-linear decrease in the TEMPOL signal intensity with a negative slope with increasing exposure time. With increasing protein concentration from 50 to 125 mg·ml^−1^, a decreased concentration of TEMPOL was investigated (Fig. 7A, C, E). The protein containing samples stored in the dark showed only small decrease of TEMPOL in comparison to the irradiated samples. The protein containing samples stored in the dark might contain radicals as well due to sample handling irradiation time prior to the measurement leading to a slightly decrease of TEMPOL. The mAb-Ι and mAb-ΙΙ showed similar results at 50 mg·ml^−1^ and 125 mg·ml^−1^ concentration. A difference can be observed when irradiating 100 mg·ml^−1^ mAb-ΙΙ solution, which results in TEMPOL signal intensity decreasing much faster than in 100 mg·ml^−1^ mAb-Ι solution. The non-linear correlation might be caused by conversion to another more inefficient photosensitizer or by radical residues damaging the chromophore, which might lead to a decreased formation of radicals or singlet oxygen. Moreover, the concentration of TEMPOL decreased as a result of the reaction. For further reactions, less TEMPOL is available, which leads to less quenching in the second order kinetics (TEMPOL interacts with radicals, leading to a decreased concentration which is available for further reaction mechanism). A linear correlation of generated “reactive species” and the formation of HMWs of protein molecules is not obvious from our data (supplementary material section, Figs. S7-S9 and Tables S6-S8). The non-linear correlation may result from a reduced occurrence of TEMPOL, since its reaction with other radicals leading to a decreased concentration (supplementary material section, Figs. S7-S9 and Tables S6-S8). However, the concentration-dependent mechanism of photochemical formation of “reactive species” may be caused by the photosensitizing compound from the mAb formulation. We suggest that the photosensitizer changes during light exposure, becoming inactive or destroyed, due to the decrease in efficiency of TEMPOL deactivation.

## 4 Conclusions

Drug formulations, including mAbs, have been found to be photosensitive when exposed to visible light λ = 400–800 nm. However, the underlying mechanism and the photoactive compounds are still unknown and have rarely been studied. The results presented here provide evidence that light-induced degradation of mAb formulations occurs through a complex mechanism which is protein-concentration-dependent. Since photodegradation increases with increasing protein concentration, it is likely that a photosensitizer is present and increases with increasing protein content. However, other photosensitizers can also be introduced to the drug product formulation through the presence of excipients, as can be deduced from the polysorbate 20 HP example. We investigated the formation of ROS upon exposure to visible light, which is accompanied by a decrease in the oxygen content in the headspace of the vial and physical and chemical changes in mAb species such as the formation of high molecular weight protein particle species, modified oxidation variants and oxidation of Met and Trp residues. A reduction of the oxygen content in the headspace using a nitrogen overlay, decreases protein oxidation.

In summary, we conclude from this study:

i. The oxygen depletion in the vial headspace is directly correlated with high molecular weight protein particle formation.
ii. The presence of nitrogen in the headspace reduces photoinduced protein degradation.
iii. The observed photoinduced protein degradation scales with protein concentration.
iv. The protein concentration dependence shows that an unknown photosensitizer contributes to the photooxidation of the protein solution and is not introduced by excipients (phosphate, trehalose).
v. Polysorbat 20 HP as excipient, however, might introduce additional chemical byproducts that act as photosensitizer, which leads to an increased photooxidation of mAbs.
vi. Radicals introduced by photooxidation of protein solutions can be monitored by electron paramagnetic resonance (EPR) spectroscopy.

## Notes

No potential competing interest was reported by the authors.

## CRediT authorship contribution statement

**Elena Hipper:** Methodology, Data curation, Formal analysis, Validation, Investigation, Writing – original draft, Writing – review & editing. **Florian Lehmann:** Data curation, Investigation, Resources. **Wolfgang Kaiser:** Formal analysis, Writing – review & editing. **Göran Hübner:** Data curation, Investigation, Writing – review & editing. **Julia Buske:** Validation, Conceptualization, Resources, Writing – review & editing. **Michaela Blech:** Validation, Resources, Investigation, Formal analysis, Methodology, Writing – review & editing. **Dariush Hinderberger:** Writing – review & editing, Supervision, Resources. **Patrick Garidel:** Funding acquisition, Project administration, Resources, Supervision, Conceptualization, Writing – review & editing.

## Declaration of Competing Interest

The authors declare that they have no known competing financial interests or personal relationships that could have appeared to influence the work reported in this paper.

## Data Availability

Data will be made available on request.

## Appendix A Supplementary data

Supplementary material Image 31
