# Supplementary material for: Protein photodegradation in the visible range? Insights into protein photooxidation with respect to protein concentration
Source: Int J Pharm X. 2022 Dec 27;5:100155. doi: 10.1016/j.ijpx.2022.100155 (PMC9926095; doi:10.1016/j.ijpx.2022.100155)
Supplement: Supplementary file 1 — Supplementary material [file mmc1.docx]

**Supplementary Materials**

Protein photodegradation in the visible range? Insights into protein photooxidation with respect to protein concentration

Elena Hipper^a,1,2^, Florian Lehmann^a,1,2^, Wolfgang Kaiser^b^, Göran Hübner^c^, Julia Buske^b^, Michaela Blech^b^, Dariush Hinderberger^a^ and Patrick Garidel^a,b*^

^a^ Institute of Chemistry, Martin-Luther-Universität Halle-Wittenberg, von-Danckelmann-Platz 4, 06120 Halle, Germany;

^b^ Boehringer Ingelheim Pharma GmbH & Co. KG, Innovation Unit, PDB-TIP, Birkendorfer Strasse 65, 88397 Biberach an der Riss, Germany;

^c^ Boehringer Ingelheim Pharma GmbH & Co. KG, Innovation Unit, ADB, Birkendorfer Strasse 65, 88397 Biberach an der Riss, Germany;

*Corresponding author

^1^ Shared first authorship.

^2^ These authors contributed equally.


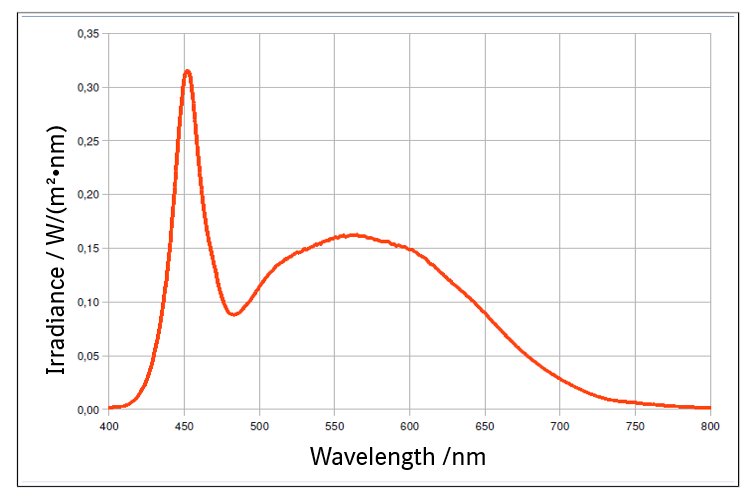


**Figure S1.** Emission spectrum of the LED (visible light-emitting diode) used for photoirradiation.

**
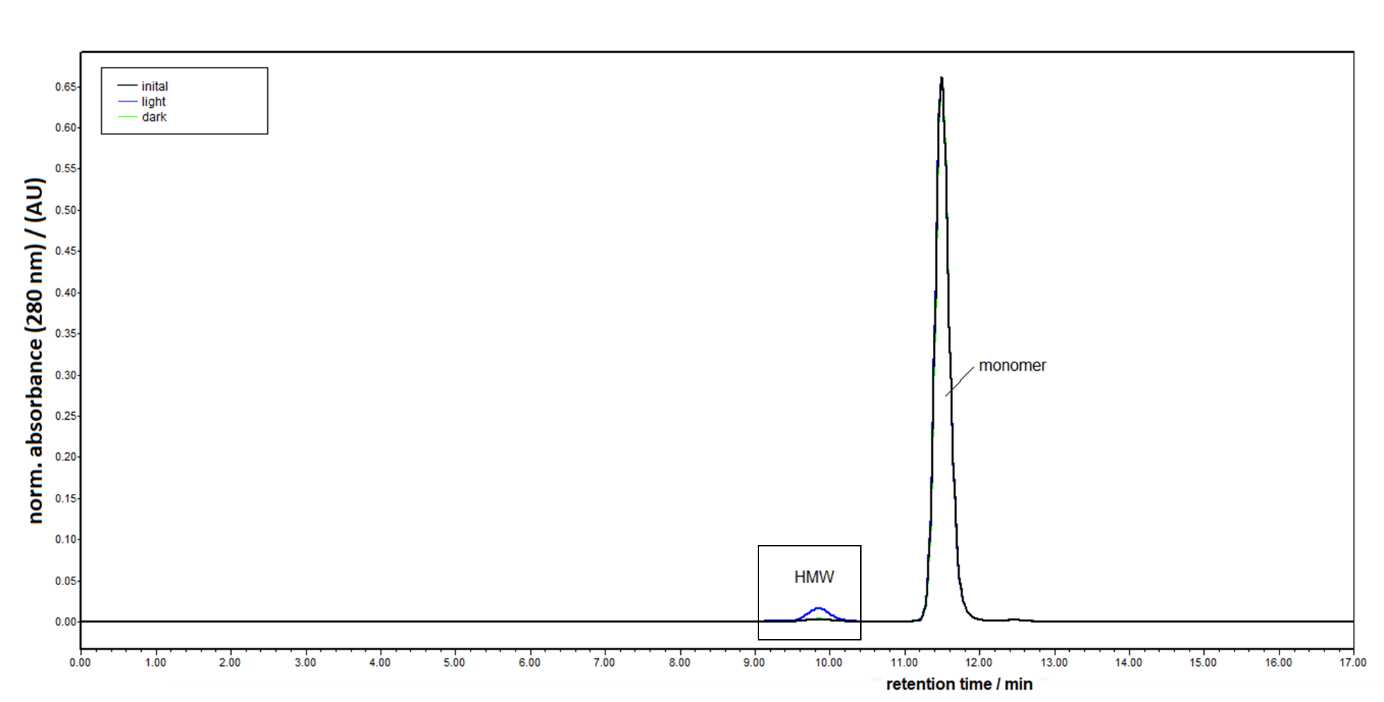
**

**Figure S2:** Exemplary chromatogram of size-exclusion chromatography of light exposed mAb. The measured absorbance signal was normalized against the main protein peak of the light protected sample (dark) for better visualization. The aggregate peak is labeled.

***
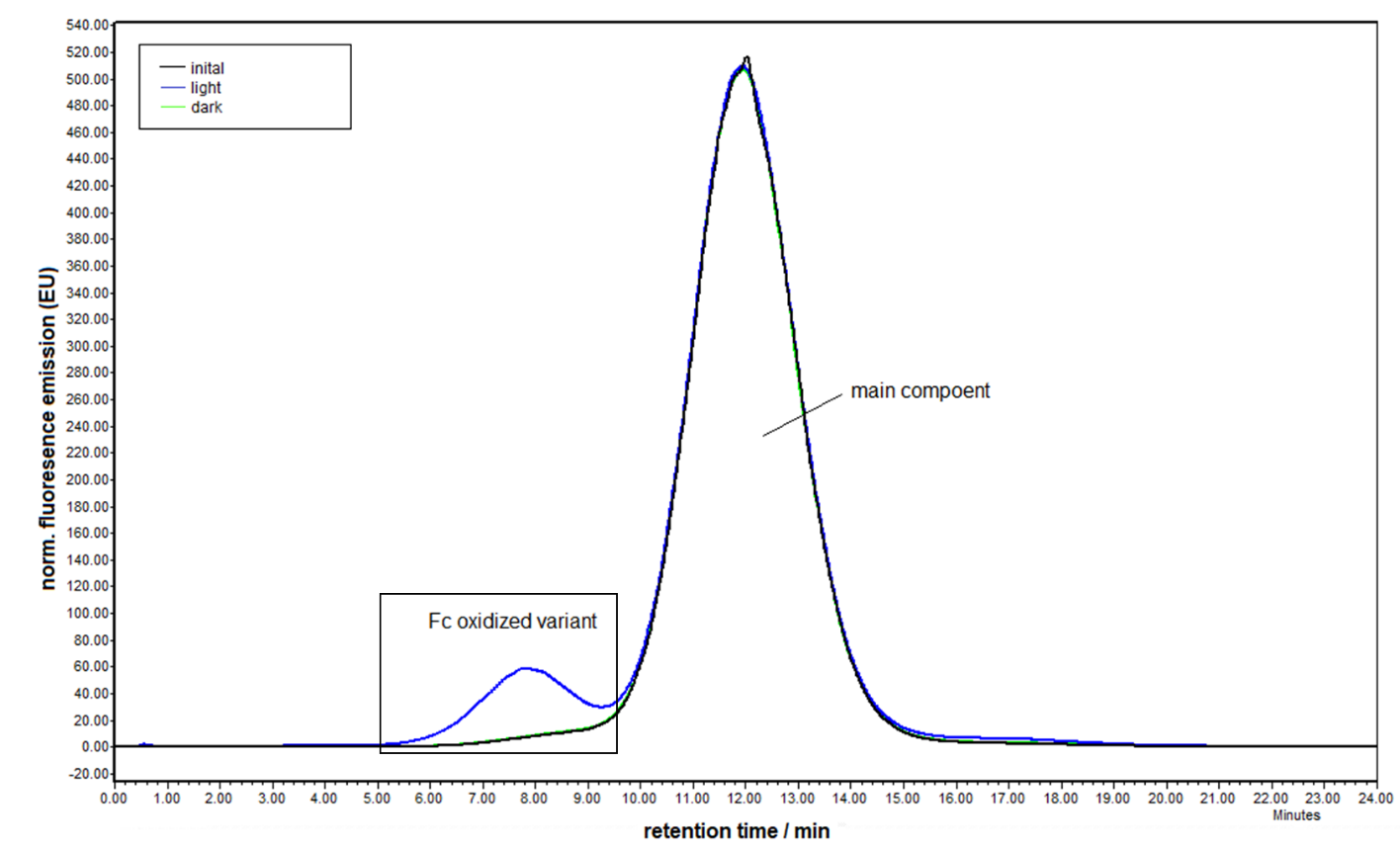
***

**Figure S3:** Exemplary chromatogram of Protein A chromatogram of light exposed mAb. The measured fluorescence signal was normalized against the main protein peak of the light protected sample (dark) for better visualization. The Fc oxidized variant group is labeled.

**
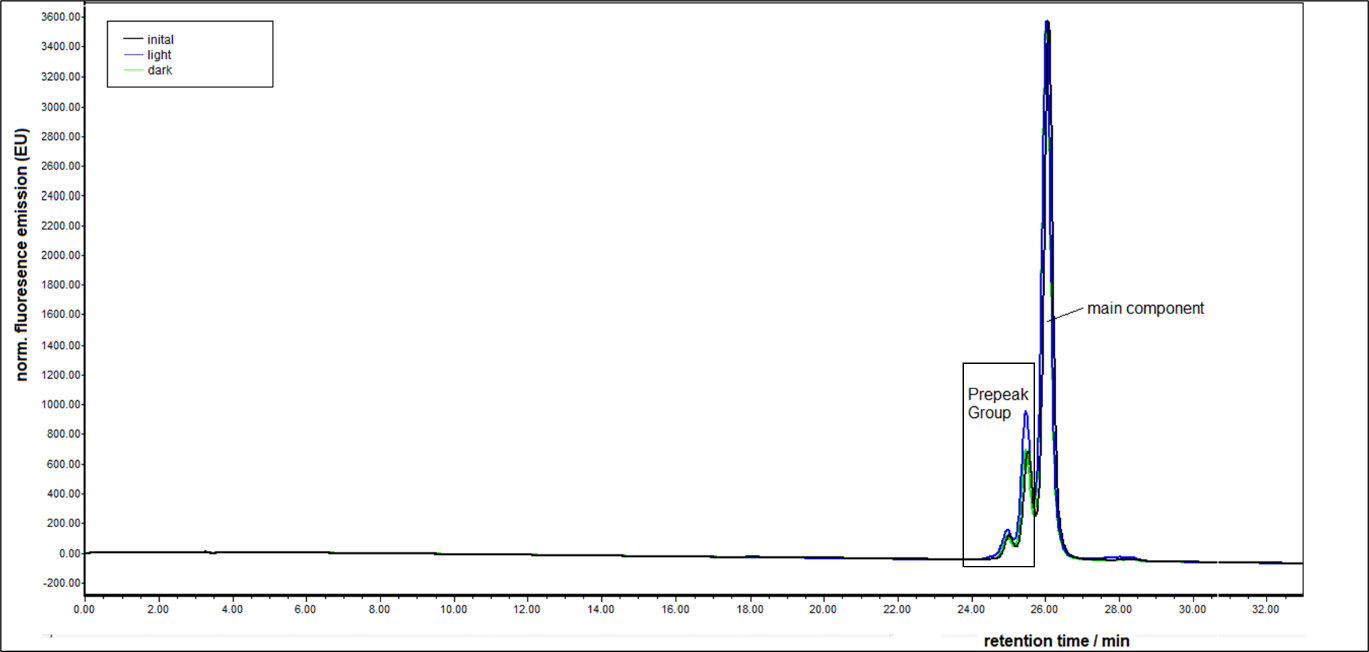
**

**Figure S4:** Exemplary chromatogram of hydrophobic interaction chromatogram of light exposed mAb. The measured fluorescence signals were normalized against the main protein peak of the light protected sample (dark) for better visualization. The prepeak group is labeled.

**Figure S5:** Dark control protein containing samples exemplary shown for mAb for the oxygen content depletion.

(**Orange**: 50 mg⋅ml^-1^, **blue**: 100 mg⋅ml^-1^ and **grey**: 150 mg⋅ml^-1^ mAb)

| Equation | y = a + b⋅x | |  |
| --- | --- | --- | --- |
| Plot | **mAb-Ι** | **mAb-ΙΙ** | **mAb-ΙΙΙ** |
| Intercept | 2.98 ± 5.16033 | -4.97 ± 6.1035 | -2.3875 ± 7.37889 |
| Slope | -0.3739 ± 0.05517 | -0.49865 ± 0.06525 | -0.451 ± 0.07888 |
| Residual Sum of Squares | 7.61E+01 | 1.06E+02 | 1.56E+02 |
| Pearson's r | -0.97892 | -0.98331 | -0.97074 |
| R-Square (COD) | 0.95828 | 0.96689 | 0.94234 |
| Adj. R-Square | 0.93742 | 0.95033 | 0.91351 |

**Table S1.** Correlation of protein concentration and oxygen content of the three mAbs at 720·10^3^ lx∙h. Belongs to Figure 2A.

| 720 · 10^3^ lx∙h | | | |
| --- | --- | --- | --- |
| Equation | **y = a + b⋅x** | | |
| Plot | **mAb-Ι** | **mAb-ΙΙ** | **mAb-ΙΙΙ** |
| Intercept | 0.002 ± 0.0099 | 0.215 ± 0.39053 | 0.119 ± 0.13731 |
| Slope | 0.00784 ± 1.0583E-4 | 0.0255 ± 0.00417 | 0.02358 ± 0.00147 |
| Residual Sum of Squares | 2.80E-04 | 0.43575 | 0.05387 |
| Pearson's r | 0.99982 | 0.97423 | 0.99615 |
| R-Square (COD) | 0.99964 | 0.94912 | 0.99231 |
| Adj. R-Square | 0.99945 | 0.92368 | 0.98846 |

**Table S2.** Correlation of protein concentration and HMW of three mAbs at 720·10^3^ lx∙h. Belongs to Figure 2B.

| 240 ·10^3^ lx∙h | | | |
| --- | --- | --- | --- |
| Equation | **y = a + b⋅x** | | |
| Plot | **mAb-Ι** | **mAb-ΙΙ** | **mAb-ΙΙΙ** |
| Intercept | 0.004 ± 0.01283 | 0.081 ± 0.09217 | 0.041 ± 0.0459 |
| Slope | 0.00338 ± 1.37113E-4 | 0.01242 ± 9.85292E-4 | 0.01012 ± 4.90714E-4 |
| Residual Sum of Squares | 4.70E-04 | 0.02427 | 0.00602 |
| Pearson's r | 0.99836 | 0.99377 | 0.99766 |
| R-Square (COD) | 0.99672 | 0.98757 | 0.99532 |
| Adj. R-Square | 0.99508 | 0.98135 | 0.99298 |

**Table S3.** Correlation of protein concentration and HMW of three mAbs at 240 ·10^3^ lx∙h. Belongs to Figure 2C.

| Equation | y = a + b⋅x | | |
| --- | --- | --- | --- |
|  | **mAb- Ι** | **mAb- ΙΙ** | **mAb- ΙΙΙ** |
| Slope | 0.02152 ± .00198 | 0.05196 ± 0.001 | 0.04913 ± 0.0038 |
| Residual Sum of Squares | 0.12482 | 0.26077 | 0.88273 |
| Pearson's r | 0.97541 | 0.99601 | 0.98226 |
| R-Square | 0.95142 | 0.99203 | 0.96483 |
| Adj. R-Square | 0.94333 | 0.9907 | 0.95897 |

**Table S4.** Correlation of decreased oxygen content and HMW of three mAbs. Belongs to Figure 3.

| 50 mg⋅ml^-1^  mAb-Ι |  | 0 | Light condition  24h with 10.000 lx | Light condition  72h with 10.000 lx | Dark control  24h | Dark control  72h |
| --- | --- | --- | --- | --- | --- | --- |
|  | HMW content / % | 2.19 | 2.35 | 2.58 | 2.18 | 2.16 |
|  | Monomer content / % | 95.18 | 94.99 | 94.77 | 95.22 | 95.20 |
|  | LMW content / % | 2.63 | 2.66 | 2.65 | 2.6 | 2.63 |
| 100 mg⋅ml^-1^  mAb-Ι | HMW content / % | 2.31 | 2.66 | 3.10 | 2.29 | 2.29 |
|  | Monomer content / % | 94.92 | 94.68 | 94.19 | 95 | 95.03 |
|  | LMW content / % | 2.78 | 2.68 | 2.71 | 2.7 | 2.68 |
| 150 mg⋅ml^-1^  mAb-Ι | HMW content / % | 2.46 | 2.96 | 3.64 | 2.45 | 2.45 |
|  | Monomer content / % | 94.78 | 94.30 | 93.62 | 94.73 | 95.01 |
|  | LMW content / % | 2.76 | 2.74 | 2.74 | 2.81 | 2.54 |

**Table S5**. Exemplary HMW, monomer and LMW content upon light exposure and mAb-Ι protein control sample stored in the dark at different concentrations measured with size-exclusion chromatography.

HMW = High Molecular Weight, LMW = Low Molecular Weight

**Figure S6.** The relative integral TEMPOL intensity evaluated from experimental EPR spectra measured at various cumulative light dosages of the buffer containing 50 µm TEMPOL. The double integral (DI) was calculated from each EPR spectrum and then normalized to the DI before light exposure (DI_0_).

**Figure S7.** EPR measurement correlated with size exclusion chromatography measurements. Correlation of decreased TEMPOL content and HMW of mAb-Ι.

| Equation | y = Intercept + B1⋅x + B2⋅x^2^ | | |
| --- | --- | --- | --- |
| Plot | **HMW** | **HMW** | **HMW** |
| Intercept | 0.93525 ± 0.40542 | 0.71524 ± 0.06124 | 0.74044 ± 0.08217 |
| B1 | 0.07386 ± 0.8299 | 0.54403 ± 0.12932 | 0.50334 ± 0.1781 |
| B2 | -0.00994 ± 0.42452 | -0.25981 ± 0.06818 | -0.24412 ± 0.0963 |
| Residual Sum of Squares | 1.22E-06 | 4.57E-07 | 1.76E-06 |
| R-Square (COD) | 0.89186 | 0.98704 | 0.96777 |
| Adj. R-Square | 0.8486 | 0.98186 | 0.95487 |

**Table S6.** EPR measurement correlated with size exclusion chromatography measurements. Correlation of TEMPOL content and HMW of mAb-Ι. Data of the Polynomial fitting.

**Figure S8.** EPR measurement correlated with size exclusion chromatography measurements. Correlation of decreased TEMPOL content and HMW of mAb-ΙΙ.

| Equation | y = Intercept + B1⋅x + B2⋅x^2^ | | |
| --- | --- | --- | --- |
| Plot | **HMW** | **HMW** | **HMW** |
| Intercept | 0.93525 ± 0.40542 | 0.71524 ± 0.06124 | 0.74044 ± 0.08217 |
| B1 | 0.07386 ± 0.8299 | 0.54403 ± 0.12932 | 0.50334 ± 0.1781 |
| B2 | -0.00994 ± 0.42452 | -0.25981 ± 0.06818 | -0.24412 ± 0.0963 |
| Residual Sum of Squares | 1.22E-06 | 4.57E-07 | 1.76E-06 |
| R-Square (COD) | 0.89186 | 0.98704 | 0.96777 |
| Adj. R-Square | 0.8486 | 0.98186 | 0.95487 |

**Table S7.** EPR measurement correlated with size exclusion chromatography measurements. Correlation of decreased TEMPOL content and HMW of mAb-ΙΙ. Data of the Polynomial fitting.

**Figure S9.** EPR measurement correlated with size exclusion chromatography measurements. Correlation of decreased TEMPOL and HMW of mAb-ΙΙΙ.

| Equation | y = Intercept + B1⋅x + B2⋅x^2^ | | |
| --- | --- | --- | --- |
| Plot | **HMW** | **HMW** | **HMW** |
| Intercept | -0.75023 ± 0.30541 | 0.28784 ± 0.24856 | 0.36818 ± 0.32247 |
| B1 | 3.52213 ± 0.6307 | 1.44421 ± 0.53027 | 1.27098 ± 0.69128 |
| B2 | -1.77215 ± 0.32548 | -0.73251 ± 0.28238 | -0.63981 ± 0.36978 |
| Residual Sum of Squares | 9.71E-07 | 5.90E-06 | 1.49E-05 |
| R-Square (COD) | 0.97382 | 0.91933 | 0.86718 |
| Adj. R-Square | 0.96334 | 0.88706 | 0.81405 |

**Table S8.** EPR measurement correlated with size exclusion chromatography measurements. Correlation of decreased TEMPOL content and HMW of mAb-ΙΙΙ. Data of the Polynomial fitting.
